# First Draft Genome of the Sable, *Martes zibellina*

**DOI:** 10.1093/gbe/evaa029

**Published:** 2020-02-14

**Authors:** Guangshuai Liu, Chao Zhao, Dongming Xu, Huanxin Zhang, Vladimir Monakhov, Shuai Shang, Xiaodong Gao, Weilai Sha, Jianzhang Ma, Wei Zhang, Xuexi Tang, Bo Li, Yan Hua, Xiaofang Cao, Zhen Liu, Honghai Zhang

**Affiliations:** e1 College of Life Science, Qufu Normal University, China; e2 State Key Laboratory of Genetic Resources and Evolution, Kunming Institute of Zoology, Chinese Academy of Sciences, Kunming, Yunnan, China; e3 College of Marine Life Science, Ocean University of China, Qingdao, Shandong, China; e4 Institute of Plant and Animal Ecology, Ural Branch, Russian Academy of Sciences, Yekaterinburg, Russia; e5 College of Biological and Environmental Engineering, Binzhou University, China; e6 College of Wildlife Resources, Northeast Forestry University, Harbin, China; e7 Novogene Bioinformatics Institute, Beijing, China

**Keywords:** *Martes zibellina*, genome assembly, adaptation, phylogeny

## Abstract

Members of genus *Martes* provide early warning signals about forest ecosystem health and are designated as a Management Indicator Species. As one of the most widespread members in *Martes*, the sable (*Martes zibellina*) is a circumboreal small predator found throughout all taiga zoogeographical zones of Eurasia and shows distinct population differentiation and morphological variations. To support further studies on striking local adaptation and population evolution, we present the first sable genome, assembled de novo from an individual originating in the Great Khingan Mountains (China). The assembled genome is 2.42 Gb, consisting of 15,814 scaffolds with a scaffold N50 of 5.20 Mb. Searches for complete Mammalia BUSCO (Benchmarking Universal Single-Copy Ortholog) gene groups found that 95.15% of the curated single-copy orthologs were assembled as complete, suggesting a high level of completeness of the genome. We totally predicted 19,413 protein-coding genes, and 0.82 Gb of repeat sequences was annotated. We also detected 1,257 olfactory receptor genes and found more functional olfactory receptor genes in sable than in other Mustelidae species, which provide a possible genetic explanation for the acute sense of smell of the sable for searching the preys under deep snow. Phylogenetic analyses revealed that the ferret (*Mustela putorius furo*) and sea otter (*Enhydra lutris*) form a clade that is sister to the sable, which was dated ∼16.4 Ma. Overall, our study provided the first reference genome for research in a broad range of areas including local adaptations, population evolution, conservation, and management for sable.

## Introduction

The sable (*Martes zibellina*) is a circumboreal species and belongs to the genus *Martes* (Mustelidae, Carnivora). Because *Martes* are very sensitive to changes in their habitats, they provide early warning signals about ecosystem health and are designated as a Management Indicator Species in national forests of some regions (Aubry et al. 2012). Sable has evolved a suite of interesting adaptive morphological associated with their cold circumboreal lifestyle, such as lustrous and silky pelage to keep warm and an excellent hearing and smelling ability to locate prey under snow (Monakhov 2011). Olfaction is one of the most important senses in most mammals and is used for finding foods, avoiding dangers, identifying mates and offspring, and identifying marked territory (Niimura and Nei 2006; Nei et al. 2008; Adipietro et al. 2012). Previous studies have shown that species-specific environmental adaptations are correlated with the number of functional and nonfunctional olfactory receptor (OR) genes retained (Hughes et al. 2018). However, to date, few studies have examined OR evolution and adaptation among Mustelidae species that display extensive ecomorphological diversity. Moreover, because the sable has evolved an excellent smelling ability to locate prey under snow (Monakhov 2011), we predict more functional OR genes in the sable than in other Mustelidae species genomes.

The Mustelidae is the most species-rich family within the mammalian order Carnivora and the diversification of the Mustelidae is a striking example of rapid adaptive radiation (Schluter 2000). As with many cases of adaptive radiation and recent speciation event, resolving the phylogenetic history within the Mustelidae, especially among genera, has been challenging. Previously, several molecular studies of the phylogenetic investigations on species within the Mustelidae were based on a limited number of mitochondrial and nuclear genes (Marmi et al. 2004; Koepfli et al. 2008; Wolsan and Sato 2010; Yu et al. 2011; Sato et al. 2012; Li et al. 2014). Taking advantage of next-generation sequencing, many genomes of Mustelidae species have been sequenced, providing us an opportunity to improve our ability to clarify the phylogenetic relationship and divergence time of this evolutionary taxon. However, no genomes are currently available for genus *Martes*.

As one of the most widespread members in *Martes*, the sable inhabits various zoogeographical zones in the mountain and plain taiga, and also coniferous and deciduous forests (Monakhov 2011). Substantial phenotypical or morphological variations (body size, fur color, and skull attributes) have been observed between genetically divergent populations of the sable (Monakhov 2011, 2015, 2016), suggesting that population differentiation associated with local adaptation may occur in different sable populations. The availability of genomic information will facilitate further studies of population structure and genomic basis of phenotype variations among different sable populations. Here, we provided the first genome assembly of the sable and demonstrated a high level of completeness of the assembly. This genome assembly provides valuable genomic resource toward studies of local adaptation, population dynamics, and conservation genomics of this ecologically important species.

## Materials and Methods

### Sample Collection

Muscle tissue for whole-genome sequencing was obtained from a single male individual (body mass: 1.1 kg, body length: 37.5 cm) from the Greater Khingan mountains (Heilongjiang Province, China). Additionally, five transcriptomic samples (heart, kidney, lung, spleen, and muscle) from the same individual were collected and stored in liquid nitrogen. Genomic DNA and total RNA were then extracted for the whole-genome and transcriptome sequencing. (See Supplementary Material online for additional details on DNA and RNA extractions, libraries construction, and sequencing.) All animal handling and experimental procedures were approved by the Animal Care and Use Committee of Qufu Normal University (Permit Number: QFNU2014-006).

### Genome Sequencing, Assembly, and Assessment

Genomic sequencing libraries with different insert sizes (230 bp, 500 bp, 2 kb, 5 kb, 10 kb, and 15 kb) were constructed and sequenced on the Illumina HiSeq 2500 platform (Illumina). The quality of raw reads was assessed using NGS QC Toolkit (Patel and Jain 2012). After filtering, the remaining high-quality data were used for de novo assembly of the sable genome.

SOAPdenovo2 (Luo et al. 2012) was employed for constructing contigs and scaffolds with the optimized parameters of “-K 41” and “-d 1” for the PREGRAPH step, “-k 41” for MAP step, and “-L 43” for SCAFF step, respectively. Briefly, contigs were first de novo assembled with short reads (insert size <2 kb). Second, all short reads (insert size <2 kb) and mate-paired reads (insert size >2 kb) were mapped onto the contigs for building scaffolds. At last, we used the GapCloser v1.12 (Luo et al. 2012) with default parameters to fill the gaps in the intrascaffolds according to paired information of PE reads and generated the final genome assembly of the sable. We then used two methods, Core Eukaryotic Genes Mapping Approach (CEGMA) (Parra et al. 2007) and Benchmarking Universal Single-Copy Orthologs (BUSCO) (Simao et al. 2015) to evaluate the genome completeness using evolutionarily informed expectations of gene content.

Five transcriptomic libraries were sequenced also on an Illumina HiSeq 2500 platform. After quality control, de novo transcriptome assembly was performed using the Trinity v2.4.0 (Haas et al. 2013) with default parameters. These transcriptome data were produced to aid the annotation process.

### Genome Annotation

The repetitive regions in sable genome were identified with a combination of homology- and de novo-based approaches. For homology-based prediction, RepeatMasker v4.0.5 with the parameter of “-nolow” and the associated RepeatProteinMask v4.0.5 (Tarailo-Graovac and Chen 2009) with the parameter of “-noLowSimple” were performed for homologous comparison by searching against the Repbase database (Bao et al. 2015). In the de novo-based approach, LTR_FINDER v1.0.5 (Xu and Wang 2007) with the parameter of “-C” and RepeatScout v1.0.5 (Price et al. 2005) and RepeatModeler v1.0.8 (Smit and Hubley 2008) tools with default parameters were used to construct a de novo candidate repeat database, by which the homolog repeats were detected using RepeatMasker. We also predicted gene structures of tRNAs, rRNAs, and other noncoding RNAs using the tools of t-RNAscan-SE (Schattner et al. 2005), BLAST (Altschul et al. 1990), and Infernal v1.2 (Nawrocki and Eddy 2013), respectively. BLAST tool was used with parameters of “-p BlastN” and “-e 1e-10.” Infernal and t-RNAscan-SE tools were used with default parameters.

We combined the homology comparison, de novo prediction, and transcriptome-based methods to predict the protein-coding genes. For homology comparison, the reference protein sequences from the Ensembl database (release 91) for six mammals (human, dog, cat, ferret, mouse, and giant panda) were aligned to the sable genome using TBlastN (Gertz et al. 2006) with an E-value cutoff of 1e-5. The potential gene structure of each alignment was then predicted using GeneWise v2.2.0 (Birney et al. 2004). For transcriptome-based annotation, the transcriptomic data were mapped onto the assembled scaffolds to identify the splice junctions using TopHat v2.1.1 (Trapnell et al. 2009) and then integrated into gene models by Cufflinks v2.2.1 (Trapnell et al. 2012). Simultaneously, we used Augustus v3.2.1 (Stanke et al. 2004), GenScan (Burge and Karlin 1997), GlimmerHMM v3.0.4 (Majoros et al. 2004), and Geneid v1.4.4 (Parra et al. 2000) with appropriate parameters to perform the de novo prediction. At last, we used EVidenceModeler v1.1.0 (Haas et al. 2008) to integrate the above prediction results and generated a nonredundant reference gene set. Functional annotation of the predicted sable genes was undertaken according to homologous searches against four databases: Nr (ftp://ftp.ncbi.nih.gov/blast/db/; last accessed May 20, 2019), Swiss-Prot (UniProt Consortium 2018), KEGG (Kanehisa et al. 2016), and InterPro (Finn et al. 2017).

### Olfactory Receptor Gene Family Analysis

We also detected OR genes in the genomes of the sable and all other six Mustelidae species that have genomic sequences publicly available. The method to identify OR genes was essentially the same as described by Niimura and Nei (2007). The relative proportions of functional and nonfunctional OR genes were compared using pairwise χ^2^ tests between the sable and other six Mustelidae species. Details of the method are provided in Supplementary Material online.

### Phylogenetic Analysis and Divergence Time Estimation

Gene families were constructed according to the OrthoMCL pipeline (Li et al. 2003). We first retrieved the protein-coding sequences that are publicly available for two Mustelidae species (sea otter, *Enhydra lutris*; ferret, *Mustela putorius furo*) at present and other six mammals (human, *Homo sapiens*; cat, *Felis catus*; dog, *Canis lupus familiaris*; giant panda, *Ailuropoda melanoleuca*; polar bear, *Ursus maritimus*; weddell seal, *Leptonychotes weddellii*) from NCBI (https://www.ncbi.nlm.nih.gov/; last accessed August 20, 2019). The consensus gene set for the above eight species and sable were filtered to retain the longest coding sequence for each gene. Protein-coding sequences for each single-copy gene family were aligned by MUSCLE v3.5 (Edgar 2004) with default parameters. Sequences were then concatenated to one supergene sequence for each species, and a pairwise distance matrix was formed. Phylogenetic inference was performed using the maximum-likelihood algorithm in RAxML v7.2.8 (Stamatakis 2006) with GTR-GAMMA substitution model deduced by jModelTest2 (Darriba et al. 2012). Statistical support for bipartitions was estimated by 1,000 rapid bootstrap replicates. The Monte Carlo Markov Chain algorithm implemented in the MCMCtree tool in PAML v4.8 (Yang 2007) was used for divergence time estimation.

## Results and Discussion

Understanding the genomic basis of key adaptations, the respective impacts of selection and drift on specific genes, and how these patterns vary across the genome are central to the study of organismal evolution (Autenrieth et al. 2018). However, without whole-genome data, these biological problems remain difficult to explore especially for nonmodel organisms. Here, we present the first de novo assembly and annotation of the whole genome of the sable based on whole-genome shotgun sequencing strategy. Genomic DNA of a male sable was sequenced to generate a total of 277.04-Gb sequencing data, corresponding to a 114.48-fold coverage of the genome (supplementary table S1, Supplementary Material online). For transcriptome sequencing, a total of 34.94-Gb sequencing data were generated (supplementary table S2, Supplementary Material online). After filtering the low-quality data, 266.80-Gb clean genomic data were used to generate a draft genome of a total length of 2.42 Gb, with a scaffold N50 of 5.20 Mb and a contig N50 of 41.68 kb (table 1 and supplementary table S3, Supplementary Material online). With a total length of 2.42 Gb and a GC content of 41.80%, the general attributes of this sable genome assembly were similar to other Carnivora genomes (supplementary table S4, Supplementary Material online). Moreover, the sequencing coverage (114.48×) and scaffold N50 (5.20 Mb) are comparable to the published high-quality Carnivora genomes assembled from high-throughput sequencing data (supplementary table S4, Supplementary Material online).

**Table 1:** Statistics of the Final Assembly of the Sable Genome

Statistics	Contigs	Scaffolds
Total length (Gb)	2.32	2.42
Sequence count	126,569	15,814
Median (bp)	7,925	2,961
Mean (bp)	18,294	153,072
N50 length (bp)	41,684	5,199,373
N90 length (bp)	8,420	758,317
Sequence count (≥2 kb)	106,733	10,744
Max length (bp)	616,201	37,060,172

We then evaluated the quality of the genome assembly with respect to base-level accuracy and genome completeness. Mapping of the short-insert sequencing data (155.58 Gb in total) to the genome scaffold indicated that >95.77% of the reads could be mapped to the assembly (supplementary table S5 and fig. S1, Supplementary Material online). The CEGMA evaluation showed that 238 (95.97%) of 248 ultraconserved eukaryotic genes were found in the assembled genome (supplementary table S6, Supplementary Material online), and BUSCO assessment showed that 3,905 (95.15%) of the 4,104 Mammalia BUSCO core genes were assembled to be complete (supplementary table S7, Supplementary Material online). Above evaluation results showed that protein-coding regions are well represented in the genome, as CEGMA and BUSCO analyses both identified a near completeness of respective core gene sets in the assembly and suggested that we have largely reconstructed the whole sable genome.

**Fig. 1.**
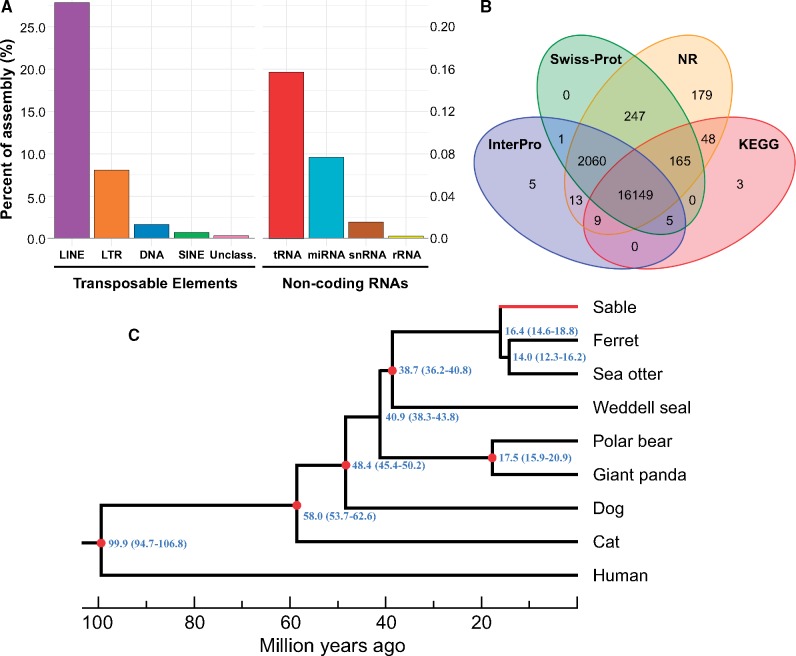
—Genome annotation and phylogenetic analysis results. (*a*) The content of transposable elements and noncoding RNA in the sable genome assembly. (*b*) Functional annotation statistics. Venn diagram illustrating distribution of high-score matches of the functional annotation in the sable genome against four public databases. (*c*) Genome-wide phylogenetic tree of the sable. We constructed the phylogenetic trees based on maximum-likelihood analyses with 7,335 one-to-one orthologous genes between the sable and other eight mammals. Five divergence times (red node) were used as the calibration points for estimating divergence time: the divergence time of Ailuropodinae and Ursinae (min = 16 Ma, max = 23 Ma), Canidae and Arctoidea (min = 44 Ma, max = 50 Ma), Pinnipedia and Musteloidea (min = 36 Ma, max = 43 Ma), Caniformia and Feliformia (min = 53 Ma, max = 63 Ma), and Primatomorpha and Carnivora (min = 95 Ma, max = 107 Ma) (Eizirik et al. 2010; Jiang et al. 2014; Hu et al. 2017). All nodes have 100% bootstrap support values. The estimated divergence times with 95% confidence intervals were shown.

Transcriptome data developed from five tissues were used for gene prediction. We obtained a total of 312,101 transcriptomic contigs with an N50 value of 2,195 bp after transcriptomic assembly (supplementary table S8, Supplementary Material online). Using a combination of de novo- and homology-based approaches, we obtained a total of 0.82 Gb of repeat elements, accounting for 33.70% of the sable genome (supplementary table S9, Supplementary Material online). The long interspersed nuclear elements were the most predominant transposable elements (28.78%) in the sable genome, followed by LTR > DNA > SINEs (fig. 1*a* and supplementary table S10, Supplementary Material online), which is consistent with findings in other mammals (Wang et al. 2017; Yang et al. 2017; Fan et al. 2018; Zhang et al. 2018). Among identified noncoding RNAs, tRNAs were the most predominant with 0.16% of the assembly (fig. 1*a* and supplementary table S11, Supplementary Material online). With a combined approach of homology-, de novo-, and transcriptome-based annotations, we identified 19,413 protein-coding genes (supplementary table S12, Supplementary Material online), similar to the ferret genome (i.e., 19,910 protein-coding genes were predicted) (Peng et al. 2014). In total, 18,884 of 19,413 (97.28%) protein-coding genes were searched within four functional databases of Swiss-Prot, KEGG, InterPro, and Nr and 16,149 genes were annotated in all four public databases (fig. 1*b* and supplementary table S13, Supplementary Material online).

We identified 1,257 OR genes in the sable genome, which included 926 intact functional genes (supplementary table S14, Supplementary Material online). The significantly more functional OR genes in sable than in other studied Mustelidae species genomes (supplementary fig. S2, Supplementary Material online; χ^2^ test *P* values for all comparisons < 0.05) may provide a possible genetic explanation for the acute sense of smell of the sable for searching the preys under deep snow. We also found extensive pseudogenization of OR genes in two otter species compared with other terrestrial Mustelidae species, consistent with the patterns of OR gene loss in other aquatic mammals (Kishida et al. 2007, 2015; Hayden et al. 2010).

To estimate species-specific and shared genes in the sable compared with eight other mammalian species, we used OrthoMCL (Li et al. 2003) to define the orthologous genes. We identified 16,770 gene families among the nine animals, in which, 50 families were specific to sable (supplementary fig. S3, Supplementary Material online). Then, we constructed a genome-wide phylogenetic tree based on the identified 7,335 one-to-one orthologous genes. The constructed phylogenetic tree confirmed previous molecular conclusions that the sable belongs to the family Mustelidae together with the ferret and sea otter (fig. 1*c*). Moreover, the ferret (subfamily Mustelinae) is closer to the clade of the sea otter (subfamily Lutrinae) than to the sable (subfamily Martinae). Based on the 4-fold degenerate codon sites on these orthologous genes, a divergence time of 16.4 Ma (95% CIs, 14.6–18.8 Ma) between sable and ferret/sea otter was derived using five calibration points (fig. 1*c*). This derived divergence time was consistent with a previous molecular-based estimate of 16.1 Ma from Yu et al. (2011). In addition, our analyses resulted in time estimates of divergence of the ferret and sea otter that agree more with those from Sato et al. (2003) than from Koepfli et al. (2008), which are less than the present results. Although the phylogenetic tree generated based on the genomic data is consistent with the current understanding of the Mustelidae phylogeny supported by previous small molecular data sets (Koepfli et al. 2008; Yu et al. 2011; Li et al. 2014), our analysis based on large-scale genomic data provided more reliable phylogenetic relationship among Mustelidae species. Moreover, we estimated the divergence time among Mustelidae species based on genomic data, which would be more accurate than the divergence time estimated based on small molecular data sets in the previous studies.

In conclusion, we present the first whole-genome assembly and annotation of the sable, and performed a genome-wide phylogenetic analysis and OR gene family analysis among Mustelidae. This sable draft genome, together with the obtained transcriptome data, provided a valuable molecular resource for studies concerning the origin, evolutionary history, and adaptation of this geographically widespread circumboreal small predator. 

## Supplementary Material

evaa029_Supplementary_Data

## Notes

**Data deposition:** Raw data from de novo sequencing and RNA-Seq are available through the NCBI Sequence Read Archive under projects PRJNA495455. Genome and transcriptome assembly data have been deposited at GenBank under the accession numbers RDFD00000000 and GHBV00000000, respectively.
