# Supplementary material for: First Draft Genome of the Sable, Martes zibellina
Source: Genome Biol Evol. 2020 Feb 14;12(3):59–65. doi: 10.1093/gbe/evaa029 (PMC7144822; doi:10.1093/gbe/evaa029)
Supplement: evaa029_Supplementary_Data [file evaa029_supplementary_data.docx]

**Supplementary Material for:**

**First draft genome of the sable, *Martes zibellina***

**Include:**

Supplementary table S1-S14; Supplementary figure S1-S3; Supplementary methods

**Table S1**. Summary statistics of genome sequencing data

|  |  | Raw data | | | Clean data^a^ | | |
| --- | --- | --- | --- | --- | --- | --- | --- |
| Library insert size (bp) | Reads length (bp) | No. of PE reads (M) | Total data (Gb) | Coverage (X)^b^ | No. of PE reads (M) | Total data (Gb) | Coverage (X)^b^ |
| 250 | 125 | 441.92 | 110.48 | 45.65 | 432.53 | 108.13 | 44.68 |
| 500 | 125 | 199.50 | 49.87 | 20.61 | 189.82 | 47.45 | 19.61 |
| 2000 | 125 | 183.64 | 45.91 | 18.97 | 172.42 | 43.10 | 17.81 |
| 5000 | 125 | 96.86 | 24.22 | 10.01 | 91.68 | 22.92 | 9.47 |
| 10000 | 125 | 121.69 | 30.42 | 12.57 | 118.46 | 29.61 | 12.24 |
| 15000 | 125 | 64.58 | 16.14 | 6.67 | 62.36 | 15.59 | 6.44 |
| Total | - | 1108.19 | 277.04 | 114.48 | 1067.27 | 266.80 | 110.25 |

Note: ^a^ Qualified reads were generated by filtering the low quality reads and adapter contamination from the raw reads; ^b^ Coverage was calculated under the assembled genome size of 2.42 Gb for the sable.

**Table S2**. Summary statistics of transcriptome sequencing data

| Tissue | Raw PE reads | Clean PE reads | Raw bases | Q20 (%) | Q30 (%) | GC content (%) |
| --- | --- | --- | --- | --- | --- | --- |
| Heart | 29,309,772 | 28,680,535 | 7,327,443,000‬ | 90.32 | 84.45 | 49.28 |
| Kidney | 27,653,838 | 26,587,962 | 6,913,459,500 | 89.56 | 82.51 | 50.89 |
| Lung | 24,308,121 | 23,664,563 | 6,077,030,250 | 93.53 | 88.88 | 50.68 |
| Spleen | 28,887,776 | 28,239,050 | 7,221,944,000 | 93.78 | 89.18 | 50.43 |
| Muscle | 29,609,303 | 28,804,737 | 7,402,325,750 | 93.95 | 89.58 | 48.76 |
| Total | 139,768,810 | 135,976,847 | 34,942,202,500 | - | - | - |

Note: Q20: percentage of bases with a Phred value of at least 20; Q30: percentage of bases with a Phred value of at least 30.

**Table S3**. Summary statistics of the genome assembly of the sable.

| Statistics | Contigs | | Scaffolds | |
| --- | --- | --- | --- | --- |
|  | Size (bp) | Number | Size (bp) | Number |
| N50 | 41,684 | 15,131 | 5,199,373 | 134 |
| N60 | 31,937 | 21,487 | 3,700,518 | 189 |
| N70 | 23,593 | 29,914 | 2,630,428 | 266 |
| N80 | 15,985 | 41,779 | 1,628,831 | 382 |
| N90 | 8,420 | 61,391 | 758,317 | 590 |
| Median (bp) | 7,925 | - | 2,961 | - |
| Mean (bp) | 18,294 | - | 153,072 | - |
| Number (Length ≥2,000) | - | 106,733 | - | 10,744 |
| Number (Length ≥100) | - | 126,569 | - | 15,814 |
| Longest sequence (bp) | 616,201 | | 37,060,172 | |
| Sequence count | 126,569 | | 15,814 | |
| Total Size | 2,315,577,164 | | 2,420,684,031 | |
| GC content | - | | 41.80 % | |

**Table S4**. Publicly available *de novo* genome assembled using next-generation sequencing data across Carnivores

| Species name | Common name | Total length (Gb) | Scaffold Number | Scaffold N50 (M) | Coverage (X) | Source |
| --- | --- | --- | --- | --- | --- | --- |
| *Acinonyx jubatus* | Cheetah | 2.38 | 14,383 | 3.12 | 75 | (Dobrynin, et al. 2015) |
| *Ailuropoda melanoleuca* | Giant panda | 2.30 | 81,467 | 1.28 | 73 | (Li, et al. 2010) |
| *Ailurus fulgens* | Red panda | 2.34 | 11,589 | 2.98 | 115 | (Hu, et al. 2017) |
| *Arctocephalus gazella* | Antarctic fur seal | 2.41 | 8,126 | 3.17 | 98 | (Humble, et al. 2016) |
| *Callorhinus ursinus* | Northern fur seal | 2.71 | 14,230 | 31.51 | 27 | N/A |
| *Enhydra lutris nereis* | Southern sea otter | 2.43 | 55,496 | 6.6 | 81 | (Beichman, et al. 2019) |
| *Gulo gulo* | wolverine | 2.42 | 47,417 | 0.18 | 75 | (Ekblom, et al. 2018) |
| *Hyaena hyaena* | Striped hyena | 2.37 | 5,760 | 2.00 | 56 | (Westbury, et al. 2018) |
| *Leptonychotes weddellii* | Weddell seal | 3.16 | 16,711 | 0.90 | 82 | N/A |
| *Mustela putorius furo* | Ferret | 2.41 | 7,783 | 9.34 | 162 | (Peng, et al. 2014) |
| *Neomonachus schauinslandi* | Hawaiian monk seal | 2.40 | 7,873 | 29.52 | 61 | N/A |
| *Neovison vison* | American mink | 2.45 | 7,175 | 6.30 | 295 | (Cai, et al. 2017) |
| *Vulpes vulpes* | Red fox | 2.50 | 676,878 | 11.80 | 94 | (Kukekova, et al. 2018) |
| *Odobenus rosmarus divergens* | Pacific walrus | 2.40 | 3,893 | 2.62 | 200 | (Foote, et al. 2015) |
| *Panthera onca* | Jaguar | 2.41 | 7,521 | 1.52 | 98 | (Figueiro, et al. 2017) |
| *Panthera pardus* | Leopard | 2.58 | 5.,377 | 21.70 | 159 | N/A |
| *Panthera tigris* | Amur tiger | 2.41 | 105,019 | 8.84 | 118 | (Cho, et al. 2013) |
| *Ursus americanus* | American black bear | 2.59 | 111,495 | 0.19 | 100 | N/A |
| *Ursus maritimus* | Polar bear | 2.31 | 23,819 | 15.94 | 101 | (Liu, et al. 2014) |

**Table S5**. Statistics of mapping results

| Statistics | | Percentage |
| --- | --- | --- |
| Reads^a^ | Mapping rate (%) | 95.77% |
| Genome^b^ | Average sequencing depth | 62.54 |
|  | Coverage at least 1X (%) | 99.96% |
|  | Coverage at least 4X (%) | 99.77% |
|  | Coverage at least 10X (%) | 98.96% |
|  | Coverage at least 20X (%) | 95.46% |

Note: ^a^ Sequencing data (totally 153.98 Gb) sequenced from short-insert sequencing data (library insert size 250 and 500) were mapped to the assembly using BWA (Li and Durbin 2010) to investigate the base-level accuracy of the assembled genome; ^b^ Coverage distribution of the sequencing data of small libraries on the assembled genome.

**Table S6**. Summary of CEGMA analysis

| Item | # Prots^a^ | % Completeness^b^ | # Total^c^ | Average^d^ | % Ortho^e^ |
| --- | --- | --- | --- | --- | --- |
| Complete | 229 | 92.34 | 313 | 1.37 | 22.71 |
| Group 1 | 59 | 89.39 | 83 | 1.41 | 23.73 |
| Group 2 | 52 | 92.86 | 60 | 1.15 | 15.38 |
| Group 3 | 58 | 95.08 | 76 | 1.31 | 18.97 |
| Group 4 | 60 | 92.31 | 94 | 1.57 | 31.67 |
| Partial | 238 | 95.97 | 425 | 1.79 | 42.86 |
| Group 1 | 63 | 95.45 | 106 | 1.68 | 41.27 |
| Group 2 | 53 | 94.64 | 75 | 1.42 | 32.08 |
| Group 3 | 60 | 98.36 | 105 | 1.75 | 43.33 |
| Group 4 | 62 | 95.38 | 139 | 2.24 | 53.23 |

Note: ^a^ number of 248 ultraconserved core eukaryotic genes (CEGs) present in the genome; ^b^ percentage of 248 ultraconserved CEGs present; ^c^ total number of CEGs present including putative orthologs; ^d^ average number of orthologs per CEG; ^e^ percentage of detected CEGs having more than 1 ortholog.

**Table S7**. Summary of BUSCO analysis by counting matches to 4,104 Mammalia single-copy orthologs

| Statistics | Count | Ratio (%) |
| --- | --- | --- |
| Complete BUSCOs^a^ | 3,905 | 95.15 |
| Complete and single-copy BUSCOs^b^ | 3,875 | 94.42 |
| Complete and duplicated BUSCOs^c^ | 30 | 0.73 |
| Fragmented BUSCOs^d^ | 102 | 2.49 |
| Missing BUSCOs^e^ | 97 | 2.36 |

Note: ^a^ The length of genes matched in the BUSCO reference group are within the expectation of the BUSCO profile match lengths; ^b^ Genes that match a single gene in the BUSCO reference group; ^c^ Genes which match genes more than once in the BUSCO reference group; ^d^ Genes only partially recovered for which the gene length exceeds the alignment length cut-off; ^e^ Not recovered genes.

**Table S8**. Statistics of the assembled sable transcriptome

| Statistics | Assembled sable transcriptome | Coding DNA sequence contigs |
| --- | --- | --- |
| Total length | 276,829,439 | 152,286,590 |
| Number of contigs | 312,101 | 51,173 |
| Median (bp) | 351 | 2,975 |
| Mean (bp) | 886 | 2,502 |
| N50 of contigs (bp) | 2,195 | 3,924 |
| N90 of contigs (bp) | 286 | 1,648 |
| Number (Length ≥ 2,000 bp) | 36,955 | 31,408 |
| Longest sequence (bp) | 34,285 | 34,285 |
| GC content (%) | 49.04 | 51.95 |

**Table S9**. Summary statistics of repeat regions in sable

| Item | Repeat Size (bp) | % of genome |
| --- | --- | --- |
| TRF | 29,975,401 | 1.24 |
| RepeatMasker | 492,558,882 | 20.35 |
| RepeatProteinMask | 212,929,508 | 8.80 |
| De novo | 674,067,920 | 27.85 |
| Total | 815,744,359 | 33.70 |

**Table S10**. Summary statistics of interspersed repeat regions in sable

| Item | Repeat TEs | | TE Proteins | | De novo | | Combined TEs | |
| --- | --- | --- | --- | --- | --- | --- | --- | --- |
|  | Length (bp) | % in Genome | Length (bp) | % in Genome | Length (bp) | % in Genome | Length (bp) | % in Genome |
| DNA elements | 31,435,268 | 1.30 | 5,264,098 | 0.22 | 5,022,394 | 0.21 | 35,251,434 | 1.46 |
| LINEs | 378,046,072 | 15.61 | 199,365,299 | 8.24 | 600,630,309 | 24.81 | 696,648,541 | 28.78 |
| LTR elements | 70,750,051 | 2.92 | 8,336,923 | 0.34 | 133,964,417 | 5.53 | 192,962,908 | 7.97 |
| SINEs | 12,724,792 | 0.53 | 0 | 0.00 | 576,355 | 0.02 | 13,232,768 | 0.55 |
| Other | 398 | 0.00 | 0 | 0.00 | 34,478 | 0.00 | 398 | 0.00 |
| Unknown | 141,470 | 0.01 | 0 | 0.00 | 4,165,268 | 0.17 | 4,305,580 | 0.18 |
| Total | 492,558,882 | 20.37 | 212,929,508 | 8.80 | 674,067,920 | 27.85 | 800,899,814 | 33.09 |

**Table S11**. Summary statistics of noncoding RNAs in sable

| Item | | Copy number | Average length (bp) | Total length (bp) | % of genome |
| --- | --- | --- | --- | --- | --- |
| miRNAs | | 18,324 | 100 | 1,839,280 | 7.60% |
| tRNAs | | 45,190 | 84 | 3,778,070 | 15.61% |
| rRNAs | 18S | 8 | 205 | 1,637 | 0.01% |
|  | 28S | 93 | 148 | 13,783 | 0.06% |
|  | 5.8S | 1 | 80 | 80 | 0.00% |
|  | 5S | 424 | 77 | 32,504 | 0.13% |
| snRNAs | CD-box | 256 | 90 | 22,921 | 0.09% |
|  | HACA-box | 277 | 137 | 37,821 | 0.16% |
|  | Splicing | 3,046 | 87 | 265,310 | 1.10% |

**Table S12**. General statistics of predicted protein-coding genes

| Gene net | | Number | Average gene length (bp) | Average CDS length (bp) | Average exons per gene | Average exon length (bp) | Average intron length (bp) |
| --- | --- | --- | --- | --- | --- | --- | --- |
| De novo | Augustus | 27,146 | 21,458 | 1,167 | 6.24 | 187 | 3,870 |
|  | GlimmerHMM | 572,093 | 3,656 | 450 | 2.43 | 185 | 2,236 |
|  | SNAP | 101,993 | 31,168 | 516 | 3.91 | 132 | 10,537 |
|  | Geneid | 52,287 | 24,401 | 812 | 4.46 | 178 | 6,625 |
|  | Genscan | 57,618 | 29,537 | 1,185 | 7.24 | 164 | 4,545 |
| Homolog | Cat | 21,454 | 22,625 | 1,355 | 7.38 | 184 | 3,336 |
|  | Dog | 31,141 | 16,701 | 1,137 | 5.53 | 206 | 3,437 |
|  | Ferret | 50,569 | 10,258 | 772 | 3.96 | 195 | 3,336 |
|  | Human | 30,226 | 17,457 | 1,095 | 5.74 | 191 | 3,454 |
|  | Mouse | 23,263 | 21,837 | 1,368 | 6.82 | 201 | 3,516 |
|  | Panda | 21,439 | 23,317 | 1,371 | 7.49 | 183 | 3,383 |
| RNA-Seq | | 65,010 | 38,362 | 1,239 | 7.25 | 171 | 3,704 |
| EvidenceModeler | | 32,422 | 18,854 | 1,056 | 5.84 | 180 | 3,676 |
| PASA2 | | 32,044 | 21,995 | 1,097 | 6.07 | 181 | 4,125 |
| Final set | | 19,413 | 35,322 | 1,557 | 9.15 | 170 | 4,145 |

**Table S13**. Functional gene annotation for sable assembly

| Item | Gene Number | Ratio (%) |
| --- | --- | --- |
| Total | 19,413 | - |
| Swiss-Prot | 18,627 | 96.04 |
| KEGG | 16,379 | 84.14 |
| InterPro | 18,242 | 93.97 |
| Nr | 18,870 | 97.20 |
| Annotated | 18,884 | 97.28 |
| Unannotated | 529 | 2.72 |

**Table S14**. Olfactory receptor (OR) gene repertoires in five Mustelidae species

| Item | Total | Intact genes | Truncated genes | Pseudogenes |
| --- | --- | --- | --- | --- |
| *Martes zibellina* | 1,257 | 926 | 19 | 312 |
| *Mustela putorius furo* | 1,254 | 846 | 20 | 388 |
| *Taxidea taxus* | 1,224 | 791 | 94 | 339 |
| *Neovison vison* | 1,176 | 781 | 8 | 387 |
| *Mellivora capensis* | 1,366 | 749 | 308 | 309 |
| *Enhydra lutris* | 1,136 | 631 | 43 | 462 |
| *Pteronura brasiliensis* | 1,149 | 626 | 112 | 411 |

Note: Truncated genes were assumed to be non-functional for this calculation.


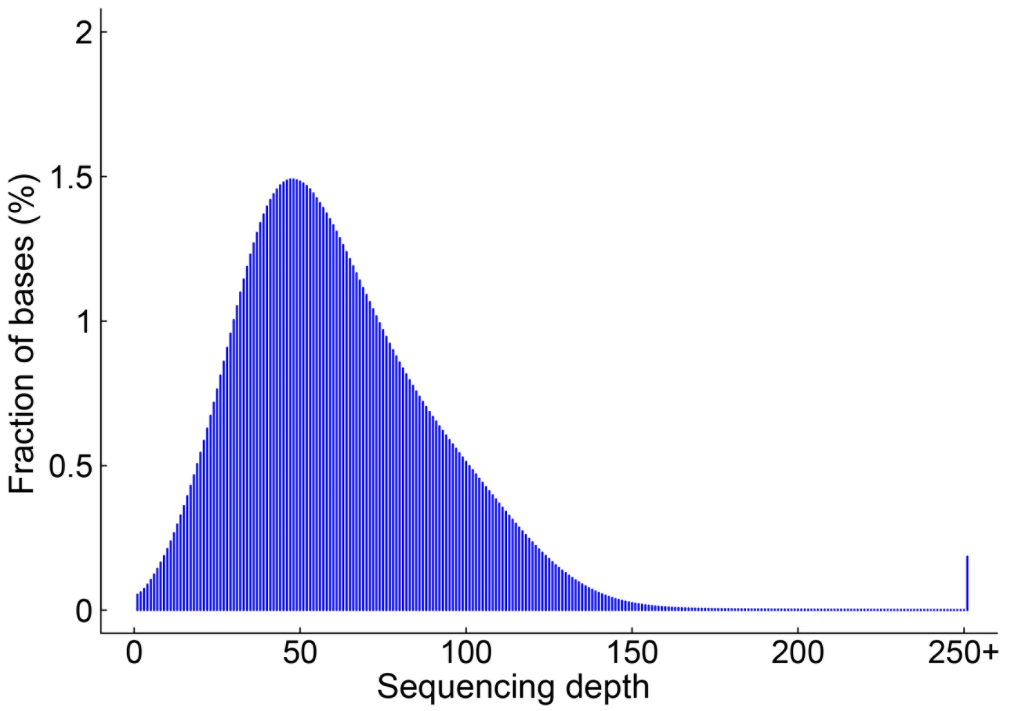


**Figure S1.** Sequencing depth of the short-insert sequencing data

The x-axis shows the sequencing depths and the y-axis shows the proportion of total bases at the given depths.


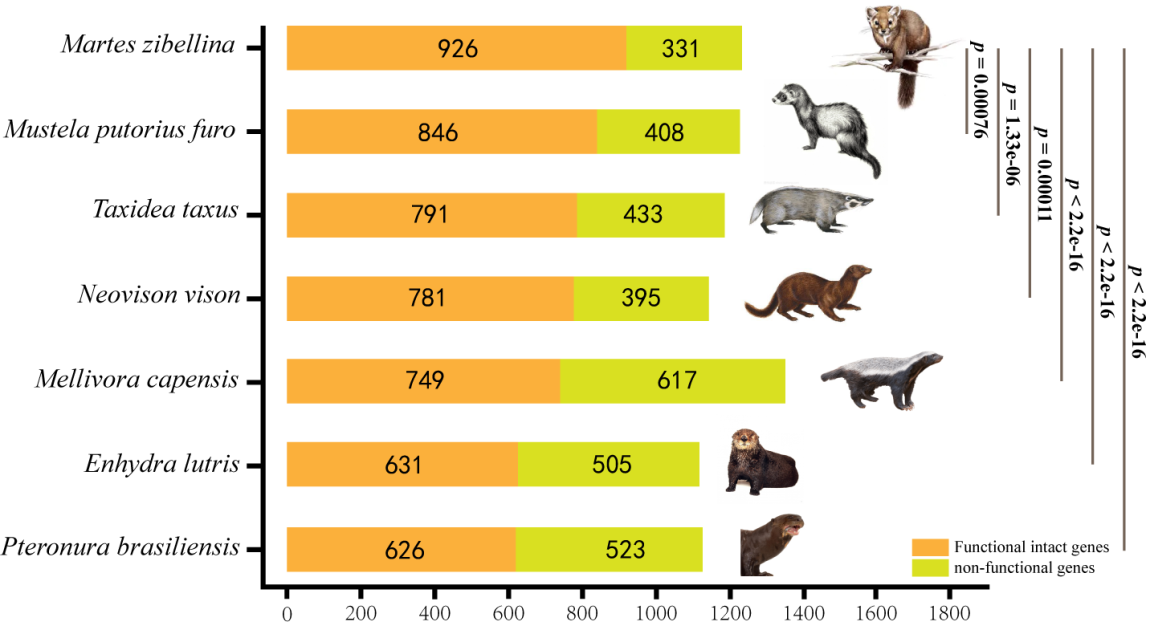


**Figure S2.** Comparison of olfactory receptor gene (OR) repertoires across seven Mustelidae species.

The sable’s OR repertoire of 926 functional genes and 331 non-functional genes had significantly more functional OR than the other six Mustelidae species (chi-squared test *p*-values for all comparisons < 0.05). See supplementary table S14 for detailed information.


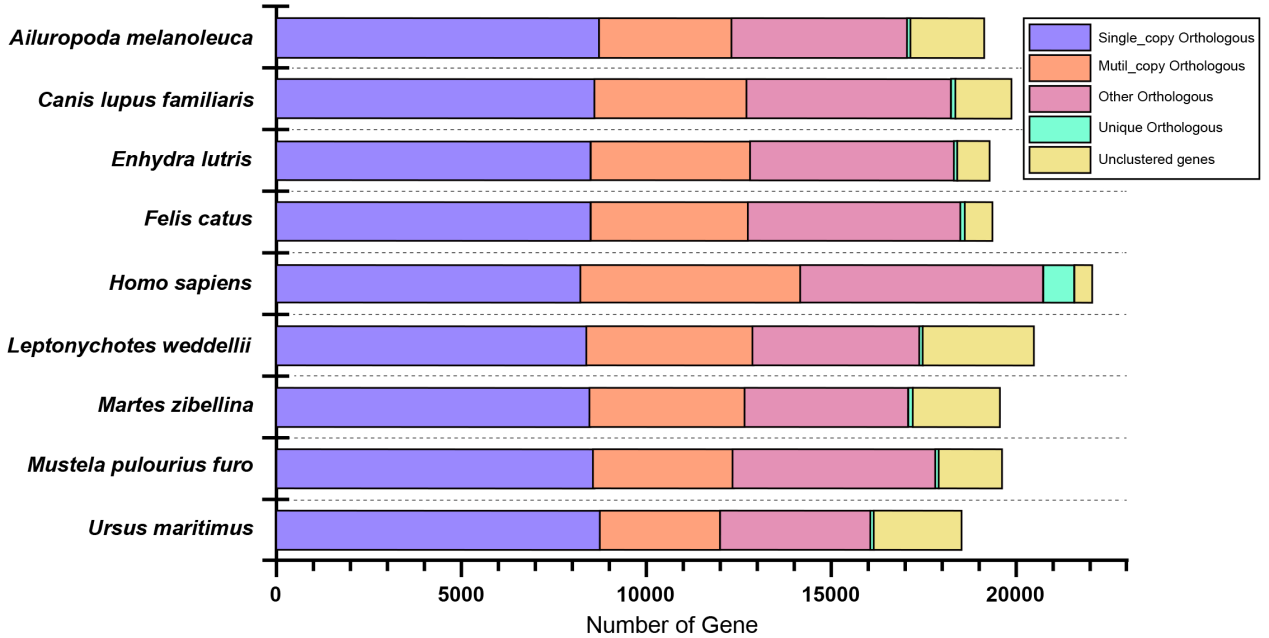


**Figure S3.** Gene family clustering of *M. zibellina* and other eight mammals.

**Supplementary methods**

**1. DNA extraction, library construction and sequencing**

Genomic DNA was extracted using the Qiagen DNeasy Blood & Tissue Kit (Qiagen, Germany) following the manufacturer’s instructions. The quality of extracted genomic DNA was checked using gel electrophoresis (1% agarose gel/50ng loading) and the total amount of genomic DNA was measured using the Qubit^®^ 2.0 Fluorometer (Life Technologies, USA). We built four short insert libraries (two for 230 bp and two for 500 bp) and seven mate pair libraries (one for 2 Kb, two for 5 Kb, two for 10 Kb and two for 15 Kb) following Illumina’s standard protocol. These libraries were sequenced in 125 bp paired-end (PE) reads on the Illumina HiSeq 2500 platform (Illumina, USA).

**2. RNA extraction, library construction and sequencing**

Total RNA was extracted from each tissue using Trizol kit (TaKaRa, Japan) according to the manufacturer’s protocol. The RNA concentration was assessed using the Qubit^®^ 2.0 Fluorometer (Life Technologies, USA). The RNA integrity was assessed using the Agilent Bioanalyzer 2100 system (Agilent, USA), and samples with an RNA Integrity Number (RIN) value greater than 8.0 were used to prepare libraries with a NEBNext Ultra™ RNA Library Prep Kit for Illumina (Illumina, USA). The transcriptomic libraries were sequenced in 125 bp PE reads on an Illumina HiSeq 2500 platform. Libraries construction and sequencing were performed at Novogene Bioinformatics Institute (Beijing, China).

**3. Identification of olfactory receptor genes**

The method to identify olfactory receptor (OR) genes was essentially the same as that described by Niimura and Nei (Niimura and Nei 2007). OR genes were classified into intact genes, truncated genes, and pseudogenes according to the definitions in Niimura (2013). The genome sequences of other 6 Mustelidae species (*Mustela putorius furo*, *Taxidea taxus*, *Neovison vison*, *Mellivora capensis*, *Enhydra lutris*, and *Pteronura brasiliensis*) were downloaded from NCBI (https://www.ncbi.nlm.nih.gov/). OR genes were mined from the genomes of the sable and other 6 Mustelidae species by the following steps:
 3.1. Conduct TBLASTN (Gertz, et al. 2006) searches with a cutoff E-value of 1e-10 against a given genome using known OR genes as queries. OR query genes were obtained from the study of Niimura (2013).
 3.2. Extract all best-hits from the genome sequences. If the same region of the genome was matched by a number of different queries, pick the one with the lowest E-value as the best-hit.
 3.3. For each of the best-hit sequences remaining after step 3.2, extend it to both directions along the genome sequence. Then, extract the longest coding sequence from an ATG codon to a stop codon. OR gene is usually a single exon gene of about 310 amino acids in length and has about 7 transmembrane regions (Niimura 2013). If the length of the sequence after extension is less than 250 amino acids, discard it. The rest will be used as candidate genes.
 3.4. Candidate genes were converted into proteins and their transmembrane structures were identified using TMHMM Server 2.0 (http://www.cbs.dtu.dk/services/TMHMM/), and those genes with 6-8 transmembrane structures were identified as intact OR genes. An intact gene putatively encodes a functional OR.
 3.5. To identify truncated OR genes and pseudogenes OR genes, the results obtained in step 3.1 were retained with the E-value below 1e-20. Extract all best-hit sequences in the same way as step 3.2, and repeat step 3.3 to obtain the candidate genes. After excluding intact OR genes identified, all remaining sequences are regarded to be truncated genes or pseudogenes.
 3.6. A truncated gene is a partial intact OR sequence. To identify truncated genes from these sequences, we extracted the sequences that did not have any nonsense or frameshift mutations and were located close (< 30 base pairs) to the contig (length ≥ 1,000bp) end. At last, all remaining sequences are regarded as OR pseudogenes, which is defined as a sequence containing any nonsense or frameshift mutations within conserved regions.
Truncated genes were assumed to be non-functional in our study. The relative proportions of functional and non-functional OR genes were compared using pairwise chi-squared tests between the sable and other 6 Mustelidae species.

**Literature Cited**

Beichman AC, et al. 2019. Aquatic adaptation and depleted diversity: a deep dive into the genomes of the sea otter and giant otter. Mol Biol Evol. 36(12):2631-2655.

Cai Z, et al. 2017. The first draft reference genome of the American mink (Neovison vison). Sci Rep. 7(1):14564.

Cho YS, et al. 2013. The tiger genome and comparative analysis with lion and snow leopard genomes. Nat Commun. 4:2433.

Dobrynin P, et al. 2015. Genomic legacy of the African cheetah, Acinonyx jubatus. Genome Biol. 16:277.

Ekblom R, et al. 2018. Genome sequencing and conservation genomics in the Scandinavian wolverine population. Conservation Biology. 32(6):1301-1312.

Figueiro HV, et al. 2017. Genome-wide signatures of complex introgression and adaptive evolution in the big cats. Sci Adv. 3(7):e1700299.

Foote AD, et al. 2015. Convergent evolution of the genomes of marine mammals. Nat Genet. 47(3):272-275.

Gertz EM, Yu YK, Agarwala R, Schaffer AA, Altschul SF. 2006. Composition-based statistics and translated nucleotide searches: improving the TBLASTN module of BLAST. BMC Biol. 4:41.

Hu Y, et al. 2017. Comparative genomics reveals convergent evolution between the bamboo-eating giant and red pandas. Proc Natl Acad Sci U S A. 114(5):1081-1086.

Humble E, et al. 2016. A draft fur seal genome provides insights into factors affecting SNP validation and how to mitigate them. Mol Ecol Resour. 16(4):909-921.

Kukekova AV, et al. 2018. Red fox genome assembly identifies genomic regions associated with tame and aggressive behaviours. Nat Ecol Evol. 2(9):1479-1491.

Li H, Durbin R 2010. Fast and accurate long-read alignment with Burrows-Wheeler transform. Bioinformatics. 26(5):589-595.

Li R, et al. 2010. The sequence and de novo assembly of the giant panda genome. Nature. 463(7279):311-317.

Liu S, et al. 2014. Population genomics reveal recent speciation and rapid evolutionary adaptation in polar bears. Cell. 157(4):785-794.

Niimura Y. 2013. Identification of chemosensory receptor genes from vertebrate genomes. Methods Mol Biol. 1068:95-105.

Niimura Y, Nei M. 2007. Extensive gains and losses of olfactory receptor genes in mammalian evolution. PLoS One. 2(8):e708.

Peng X, et al. 2014. The draft genome sequence of the ferret (Mustela putorius furo) facilitates study of human respiratory disease. Nat Biotechnol. 32(12):1250-1255.

Westbury MV, et al. 2018. Extended and continuous decline in effective population size results in low genomic diversity in the world's rarest hyena species, the brown hyena. Mol Biol Evol. 35(5):1225-1237.
